# FastSurfer-Based Brain Morphometry and Machine-Learning Classification Across the Alzheimer’s Disease Spectrum

**DOI:** 10.3390/brainsci16090980

**Published:** 2026-09-16

**Authors:** Sinem Nur Altun, Hurriyet Cetinok

**Affiliations:** Department of Anatomy, Faculty of Medicine, Istanbul Atlas University, 34403 Istanbul, Turkey; sinemaltunw@gmail.com

**Keywords:** Alzheimer’s disease, mild cognitive impairment, FastSurfer, hippocampus, morphometry, machine learning

## Abstract

**Background**: This study aimed to quantitatively assess structural changes in the hippocampus, amygdala, entorhinal cortex, lateral ventricles, precuneus, and posterior cingulate using deep-learning-based FastSurfer morphometry and evaluate their contribution to classification across the Alzheimer’s disease (AD) spectrum. **Methods**: Three-dimensional T1-weighted MRI data from the AD Neuroimaging Initiative (ADNI) were analyzed using FastSurfer. The study included 791 participants (425 female and 366 male): 365 cognitively normal (CN), 273 with mild cognitive impairment (MCI), and 153 with AD. Morphometric measures included hippocampal and amygdala volumes, lateral ventricular volume, entorhinal cortical thickness and surface area, and precuneus and posterior cingulate thickness and folding index. Volumetric measures were normalized to estimated total intracranial volume (eTIV). Principal morphometric comparisons were additionally adjusted for age, sex, and education. Six machine-learning classifiers were evaluated using participant-level training (*n* = 632) and held-out test (*n* = 159) sets, with an additional MMSE-ablation analysis. **Results**: Hippocampal and amygdala volumes were significantly lower and lateral ventricular volume was significantly higher in AD than in CN (all *p* < 0.001), with these differences persisting after eTIV normalization and remaining significant after adjustment for age, sex, and education (all adjusted *p* < 0.001). MCI generally showed intermediate volumetric values between CN and AD. Mini-Mental State Examination (MMSE) scores correlated positively with total hippocampal (*r* = 0.495) and amygdala (*r* = 0.462) volumes and negatively with lateral ventricular volume (*r* = −0.256) (all *p* < 0.001). For three-class CN/MCI/AD classification, Logistic Regression with ElasticNet achieved a mean macro-F1 score of 0.706 ± 0.017 in five-fold cross-validation and a held-out macro-F1 score of 0.705 with MMSE included. Excluding MMSE reduced the held-out macro-F1 score to 0.531 and ROC-AUC to 0.735, compared with 0.857 when MMSE was included. Performance was higher for binary CN-versus-AD classification, with a held-out test macro-F1 score of 0.919 and ROC-AUC of 0.990. **Conclusions**: FastSurfer-based morphometry demonstrated medial temporal atrophy and lateral ventricular enlargement across the CN–MCI–AD spectrum, with the principal volumetric differences remaining robust after adjustment for age, sex, and education. The intermediate morphometric profile of MCI and the associations between medial temporal volumes and cognitive performance support the relevance of these structural measures. Machine-learning performance improved substantially when MMSE was incorporated, indicating that the combined models reflect integrated morphometric, demographic, genetic, and cognitive information rather than morphometry alone. External validation in independent cohorts is required before clinical application.

## 1. Introduction

Dementia is characterized by cognitive decline that interferes with activities of daily living. Alzheimer’s disease (AD), the most common form of dementia, is a progressive neurodegenerative disorder characterized by impairments in memory, language, attention, comprehension, and reasoning [1]. At the biological level, AD is characterized by the accumulation of amyloid-β and pathological tau, accompanied by progressive synaptic dysfunction and neurodegeneration. These processes ultimately produce measurable structural alterations in brain regions involved in memory and higher-order cognitive functions [2,3,4]. Consequently, structural neuroimaging has become an important component of the quantitative characterization of AD-related neurodegeneration.

Structural changes in early AD are particularly prominent in the medial temporal lobe. The hippocampus plays a central role in episodic memory and spatial processing, while the amygdala is involved in emotional memory and regulation. The entorhinal cortex, a major pathway between the neocortex and hippocampus, is among the earliest regions affected according to Braak staging [2]. Progressive gray matter loss is accompanied by lateral ventricular enlargement, an indirect marker of cerebral atrophy. Structural and metabolic alterations also occur in the precuneus and posterior cingulate gyrus, key components of the default mode network [2,3,4]. These regions may therefore provide complementary morphometric information across the clinical spectrum from CN aging through mild cognitive impairment (MCI) to AD. Simultaneous evaluation of medial temporal volumes, cortical thickness, surface morphology, and ventricular enlargement may capture different aspects of the neurodegenerative process that are not fully represented by a single anatomical measure.

Magnetic resonance imaging (MRI) enables quantitative assessment of regional brain atrophy without exposure to ionizing radiation and is widely used to characterize structural changes associated with AD [3]. Traditional manual or semi-automated segmentation approaches, however, can be time-consuming and susceptible to operator-dependent variability, limiting their scalability in large neuroimaging datasets. Automated image-analysis pipelines provide a reproducible alternative for extracting regional volumes and cortical measures from three-dimensional T1-weighted MRI. FastSurfer, a deep learning-based framework, substantially reduces processing time compared with conventional FreeSurfer workflows while providing reproducible measures of brain volume, cortical thickness, surface area, and gyrification [5]. Its computational efficiency is particularly relevant for large multicenter datasets such as the AD Neuroimaging Initiative (ADNI), in which hundreds or thousands of MRI examinations may require standardized morphometric processing.

Artificial intelligence and machine-learning approaches have increasingly been applied to structural MRI data to identify multivariate patterns associated with cognitive impairment and AD [6,7]. Although binary discrimination between CN individuals and patients with established AD can achieve high performance, differentiation across the broader CN–MCI–AD spectrum remains more challenging because MCI represents a clinically and biologically heterogeneous intermediate state. Combining morphometric measures with demographic, cognitive, and genetic variables may therefore provide additional discriminatory information beyond structural MRI features alone. Previous studies have investigated cortical thickness, hippocampal morphology, volumetry, and deep-learning-based classification in AD and MCI [6,7].

However, studies integrating FastSurfer-based assessment of medial temporal structures and lateral ventricles with estimated total intracranial volume (eTIV) normalization, ratio-based measures, cortical morphometry, and multimodel classification remain relatively limited. Multicenter datasets such as ADNI provide a suitable framework for investigating whether the integrated assessment of medial temporal atrophy, cortical alterations, and ventricular enlargement provides complementary information for machine-learning-based classification across the CN–MCI–AD spectrum [8]. Evaluation within the same analytical framework also enables direct comparison of the relative discriminatory information provided by morphometric features alone and by models incorporating additional demographic and clinical/genetic variables.

Accordingly, the present study aimed to quantitatively evaluate hippocampal and amygdala volumes, lateral ventricular enlargement, entorhinal cortical thickness and surface area, and precuneus and posterior cingulate cortical measures using FastSurfer-based morphometry; characterize structural differences among CN, MCI, and AD groups; examine associations between selected morphometric measures and cognitive performance; and compare machine-learning models for three-class CN/MCI/AD and binary CN-versus-AD classification using morphometric features alone and in combination with demographic and clinical/genetic variables.

## 2. Materials and Methods

### 2.1. Ethical Approval

The study was approved by the Ethics Committee of Istanbul Atlas University Medical Faculty (Approval number: E-22686390-050.99-72694; Date: 28 July 2025) and conducted in accordance with the Declaration of Helsinki. Due to the retrospective design of the study and the use of anonymized patient data, the requirement for informed consent was waived.

### 2.2. Study Design and Participants

This retrospective cross-sectional study used structural magnetic resonance imaging (MRI), demographic, and cognitive data obtained from the ADNI database [9,10]. ADNI is a multicenter longitudinal initiative designed to investigate the progression of AD using clinical, imaging, and biomarker data. ADNI was launched in 2003 as a public-private partnership, led by Principal Investigator Michael W. Weiner, MD. The primary goal of ADNI has been to test whether serial magnetic resonance imaging (MRI), positron emission tomography (PET), other biological markers, and clinical and neuropsychological assessment can be combined to measure the progression of MCI and early AD. Access to the ADNI database was approved on 29 April 2025, and data retrieval from the LONI Image and Data Archive (IDA) was initiated on 1 August 2025. Participants were classified according to ADNI diagnostic information as CN, MCI, or AD. Morphometric analyses were conducted at the Department of Anatomy, Faculty of Medicine, Istanbul Atlas University.

A total of 796 participants with available three-dimensional (3D) T1-weighted structural MRI data and morphometric processing outputs were initially screened. Five participants without diagnostic information were excluded, resulting in a final analytical cohort of 791 participants: 365 CN, 273 MCI, and 153 AD. Demographic and clinical data were obtained from standard ADNI datasets, including PTDEMOG, DXSUM, Mini-Mental State Examination (MMSE), APOERES, ADNIMERGE, MRIMETA, and MRI3META.

Participants were included if diagnostic information, cognitive assessment data, and technically adequate 3D T1-weighted MRI scans suitable for morphometric analysis were available. Participants with major neurological or psychiatric disorders, severe cerebrovascular disease, intracranial mass lesions, significant head trauma, or MRI scans of insufficient technical quality were excluded. The CN group consisted of participants without MCI or dementia.

All data were obtained in anonymized form under the ADNI data-use agreement, and there was no direct contact with participants.

### 2.3. MRI Data Acquisition

**Figure 1:**
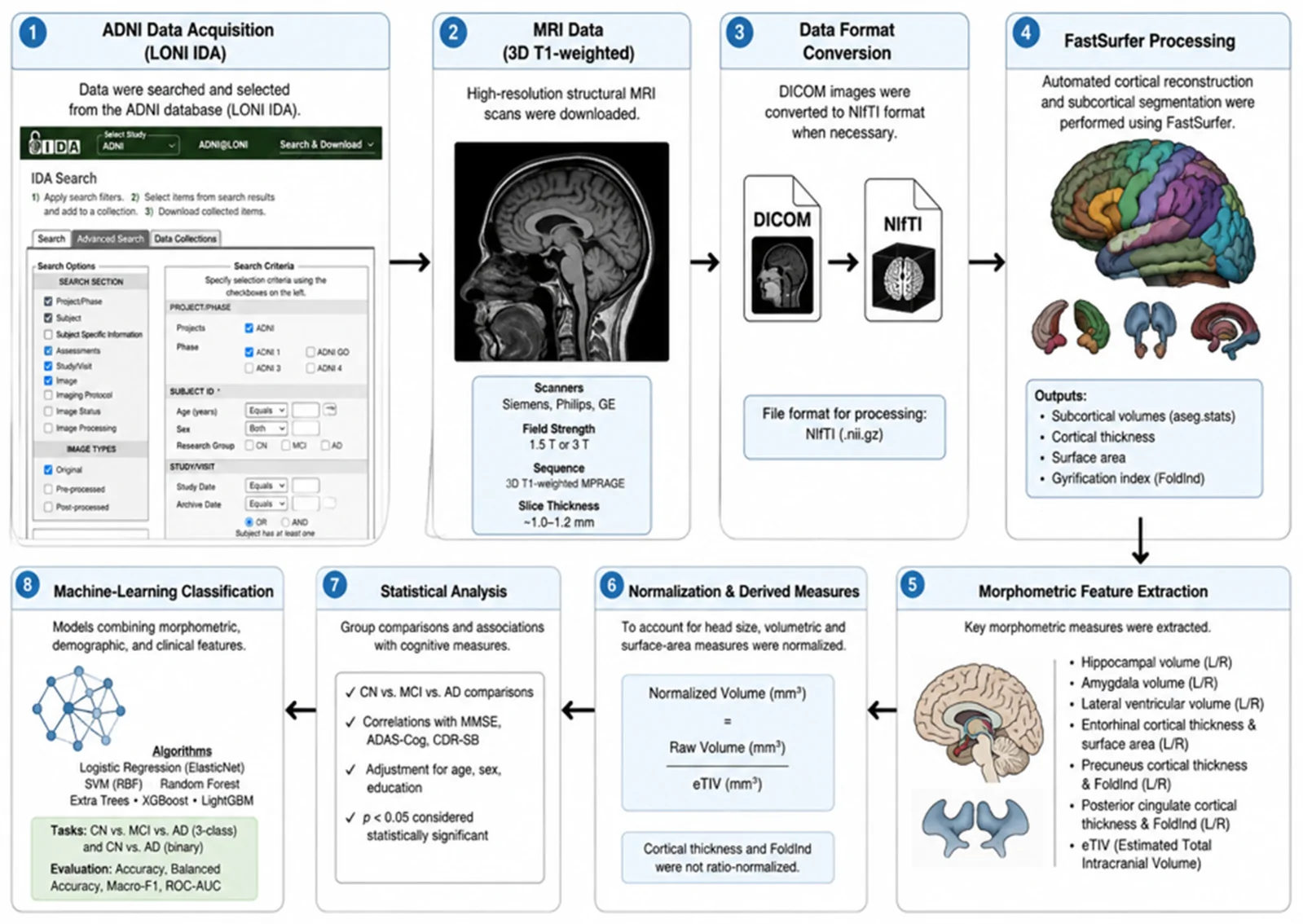
Overview of the study workflow. (1) ADNI data acquisition: eligible participants and structural MRI data were identified and retrieved from the Alzheimer’s Disease Neuroimaging Initiative (ADNI) LONI Image and Data Archive (IDA). (2) MRI data selection: high-resolution three-dimensional T1-weighted structural MRI scans acquired using Siemens, Philips, or GE scanners at 1.5 or 3 T were selected. (3) Data-format preparation: DICOM images were converted to NIfTI format when necessary. (4) FastSurfer processing: automated cortical reconstruction and subcortical segmentation were performed using the FastSurfer pipeline. (5) Morphometric feature extraction: hippocampal, amygdala, and lateral ventricular volumes; entorhinal cortical thickness and surface area; precuneus and posterior cingulate cortical thickness and FoldInd; and eTIV were extracted. (6) Normalization and derived measures: volumetric measures were normalized to eTIV, and predefined structural ratios were calculated; cortical thickness and FoldInd were not ratio-normalized. (7) Statistical analysis: unadjusted and age-, sex-, and education-adjusted group comparisons across CN, MCI, and AD were performed, and associations between selected morphometric measures and cognitive performance were evaluated. (8) Machine-learning classification: six classifiers were evaluated for three-class CN/MCI/AD and binary CN-versus-AD classification using morphometric features alone and in combination with demographic variables, including age, sex, and education, and available clinical/genetic variables.

Structural MRI data were retrieved from the LONI Image and Data Archive (IDA) according to the ADNI imaging protocol (Figure 1). As ADNI is a multicenter initiative, images were acquired using Siemens, Philips, or GE scanners at magnetic field strengths of 1.5 or 3 T. The primary structural sequence was sagittal 3D T1-weighted magnetization-prepared rapid gradient echo (MPRAGE), with an approximate slice thickness of 1.0–1.2 mm. ADNI-preprocessed volumetric scans with gradient non-linearity correction were preferentially used to minimize scanner-related variability. MRI data were acquired across multiple ADNI sites using Siemens, Philips, and GE scanners at 1.5 or 3 T. To reduce acquisition-related variability, standardized ADNI acquisition procedures were used, and ADNI-preprocessed scans, including gradient nonlinearity-corrected images when available, were preferentially selected. Scanner manufacturer and field strength were not included as covariates in the statistical models, and no additional harmonization procedure was applied.

Images were downloaded in DICOM or compressed NIfTI format. When necessary, DICOM images were converted to NIfTI (.nii.gz) using dcm2niix before morphometric processing.

### 2.4. Fast-Surfer-Based Morphometric Analysis

Automated cortical reconstruction and subcortical segmentation were performed using FastSurfer, a deep learning-based neuroimaging pipeline. This approach was used to obtain standardized morphometric measurements while minimizing operator-dependent variability and processing time.

Three-dimensional T1-weighted images were processed through the FastSurfer pipeline. Subcortical volumes were extracted from aseg.stats outputs. Cortical measures were extracted from the hemisphere-specific FastSurfer surface-statistics files (lh.aparc.DKTatlas.mapped.stats and rh.aparc.DKTatlas.mapped.stats). For the predefined entorhinal, precuneus, and posteriorcingulate regions, cortical thickness was obtained from the ThickAvg field, surface area from SurfArea, and folding index from FoldInd. The ThickAvg field represents the regional mean cortical thickness generated by the surface-analysis pipeline; therefore, no additional averaging across vertices was applied to the hemisphere-specific regional values and left and right hemispheres were analyzed separately. The cortical parcellation and relevant processing/statistics outputs were checked for technical consistency. When corresponding baseline measurements were available in ADNIMERGE, FastSurfer-derived hippocampal, ventricular, and intracranial volume measures were cross-checked against these data as part of quality control. Cases outside the predefined acceptable range were reprocessed or excluded.

### 2.5. Morphometric Measures

The morphometric variables were selected to represent medial temporal atrophy, ventricular enlargement, and cortical changes associated with AD. The primary measures included left and right hippocampal volumes (mm^3^), left and right amygdala volumes (mm^3^), left and right lateral ventricular volumes (mm^3^), entorhinal cortical thickness (mm) and surface area (mm^2^), precuneus cortical thickness and FoldInd, posterior cingulate cortical thickness and FoldInd, and eTIV.

For bilateral structures, total hippocampal, amygdala, and lateral ventricular volumes were calculated by summing the corresponding left and right measurements. Additional structural ratios, including total hippocampus/total amygdala, total lateral ventricle/total hippocampus, total lateral ventricle/total amygdala, eTIV/total hippocampus, and eTIV/total amygdala, were calculated for comparative analyses.

To account for differences in head size, hippocampal, amygdala, and lateral ventricular volumes were normalized to eTIV and expressed as ratios multiplied by 1000. Cortical thickness and FoldInd measures were not normalized to eTIV.

### 2.6. Clinical and Cognitive Measures

The MMSE was the primary cognitive measure and was available for all 791 participants. MMSE scores were used to characterize cognitive status and examine associations between cognitive performance and selected morphometric measures. ADAS-Cog and Clinical Dementia Rating–Sum of Boxes (CDR-SB) data were obtained from ADNI when available. Demographic and clinical variables, including age, sex, education, and APOE genotype when available, were also considered for machine-learning analyses.

### 2.7. Statistical Analysis

Statistical analyses were performed using IBM SPSS Statistics version 27.0. Continuous variables were summarized as mean and standard deviation and, where appropriate, median and range; categorical variables were presented as frequencies and percentages. Distributional normality of continuous variables was assessed using the Kolmogorov–Smirnov test. A *p* value > 0.05 was considered consistent with no statistically significant departure from normality. Variables meeting the normality assumption were analyzed using parametric tests, whereas variables showing significant deviation from normality were evaluated using the corresponding non-parametric tests.

For comparisons between the AD and CN groups, continuous variables were analyzed using the independent-samples *t*-test or Mann–Whitney U test according to their distribution. Comparisons of selected morphometric and cognitive variables across the CN, MCI, and AD groups were performed using the Kruskal–Wallis test. Categorical variables were compared using the chi-square test where appropriate.

Initial group-level morphometric comparisons were performed without covariate adjustment. Volumetric measures were additionally normalized to eTIV to account for interindividual variation in head size. To evaluate whether the principal morphometric differences were independent of demographic differences among the diagnostic groups, general linear model/ANCOVA analyses were subsequently performed with diagnostic group as the main factor and age, sex, and years of education as covariates.

Associations between MMSE scores and selected morphometric measures, including total hippocampal, total amygdala, total lateral ventricular, eTIV-normalized hippocampal volume, and entorhinal cortical thickness, were evaluated using Spearman rank correlation analysis.

For the adjusted analyses, volumetric outcomes included participants with complete morphometric data (*n* = 789), whereas cortical-thickness analyses included all 791 participants. Because lateral ventricular measures showed right-skewed distributions, total lateral ventricular volume and the corresponding eTIV-normalized ventricular measure were logarithmically transformed before inclusion in the adjusted models. Adjusted overall diagnostic-group effects and diagnostic-group contrasts were evaluated.

All statistical tests were two-sided, and *p* < 0.05 was considered statistically significant.

### 2.8. Machine-Learning Analysis

Machine-learning analyses were conducted in Python (version 3.9) using scikit-learn, XGBoost, and LightGBM. The unit of analysis was the individual participant to prevent multiple observations from the same participant from being treated as independent observations. The dataset was randomly divided at the participant level into a training set (*n* = 632; approximately 80%) and a held-out test set (*n* = 159; approximately 20%), using a random seed of 42.

The candidate classifiers included Logistic Regression with ElasticNet regularization, support vector machine with a radial basis function kernel, Random Forest, Extra Trees, XGBoost, and LightGBM. Two feature families were evaluated: (i) FastSurfer morphometric measures only and (ii) morphometric measures combined with demographic and clinical/genetic variables (age, sex, education, MMSE, and APOE genotype when available). Volumetric features were normalized to eTIV where appropriate, whereas cortical thickness and FoldInd measures were not ratio-normalized. APOE genotype was missing in approximately 9.5% of the training set and was handled by median/mode imputation within the training folds.

The primary classification task was three-class discrimination among CN, MCI, and AD, as this approach reflects the clinically relevant spectrum from normal cognition through intermediate impairment to established AD and represents a more challenging classification problem. Binary CN-versus-AD classification was additionally evaluated as a secondary analysis to assess discrimination between the two diagnostic extremes and provide a benchmark for comparison with the three-class task. Both the original training data and scenarios incorporating the Synthetic Minority Over-sampling Technique (SMOTE) were examined; SMOTE was applied only within the training folds to prevent information leakage. Model hyperparameters were optimized using five-fold cross-validation within the training data. Performance was evaluated using accuracy, balanced accuracy, macro-F1 score, and receiver operating characteristic area under the curve (ROC-AUC). For multiclass classification, ROC-AUC was calculated using a one-vs-rest approach. The held-out test set (*n* = 159) remained completely separate from model development and was used only for final held-out performance evaluation.

To specifically evaluate the contribution of MMSE to classification performance, an ablation analysis was additionally performed using three feature sets: (1) morphometric features alone; (2) morphometric, demographic, and genetic features without MMSE; and (3) the same combined feature set including MMSE. The same participant-level stratified train/test split (80%/20%; random seed = 42) was retained across all comparisons. For the ablation analysis, missing predictor values were imputed using medians estimated exclusively from the training data, continuous predictors were standardized using parameters derived from the training data, and APOE genotype was encoded as the number of ε4 alleles (0, 1, or 2). The same ElasticNet-regularized logistic regression framework was applied to all three feature sets to permit direct comparison. Performance on the held-out test set was summarized using accuracy, balanced accuracy, macro-F1, and ROC-AUC. No information from the held-out test set was used for imputation, scaling, model fitting, or hyperparameter optimization.

## 3. Results

Unless otherwise specified, all numerical values reported in Section 3 and Table 1, Table 2, Table 3, Table 4 and Table 5 were derived from the analyses performed in the present study using the study cohort; values from previously published studies are not included as original study results.

### 3.1. Demographic and Clinical Characteristics

A total of 791 participants were included: 365 CN, 273 MCI, and 153 AD. Mean age increased across the diagnostic groups, from 71.82 ± 6.88 years in CN to 73.51 ± 7.39 years in MCI and 75.45 ± 7.82 years in AD. The female/male distribution was 224/141 in the CN group, 127/146 in the MCI group, and 74/79 in the AD group. Mean years of education were 16.47 ± 2.39, 15.90 ± 3.03, and 15.37 ± 2.62 years in the CN, MCI, and AD groups, respectively. Mean MMSE scores progressively decreased across the groups (CN: 29.13 ± 1.13; MCI: 27.43 ± 1.91; AD: 22.81 ± 2.54). Demographic and clinical characteristics are summarized in Table 1.

Among participants with AD, 63 (41.2%) had MMSE scores of 24–30, 81 (52.9%) had scores of 20–23, and 9 (5.9%) had scores of 10–19. The mean MMSE score in the AD group was 22.8 ± 2.5.

**Table 1:** Demographic and clinical characteristics of the study groups.

Characteristic	CN (*n* = 365)	MCI (*n* = 273)	AD (*n* = 153)
Age, years	71.82 ± 6.88 (53.5–89.9)	73.51 ± 7.39 (56.0–89.0)	75.45 ± 7.82 (55.2–91.0)
Sex, female/male	224/141	127/146	74/79
Education, years	16.47 ± 2.39 (6–20)	15.90 ± 3.03 (4–20)	15.37 ± 2.62 (7–20)
MMSE score	29.13 ± 1.13 (24–30)	27.43 ± 1.91 (21–30)	22.81 ± 2.54 (11–30)

Values are mean ± SD (range) or counts. CN, cognitively normal; MCI, mild cognitive impairment; AD, Alzheimer’s disease; MMSE, Mini-Mental State Examination.

### 3.2. Morphometric Findings

Compared with CN participants, those with AD had significantly lower left and right hippocampal volumes and left and right amygdala volumes (all *p* < 0.001). In contrast, left and right lateral ventricular volumes were significantly higher in AD (both *p* < 0.001). eTIV did not differ significantly between the groups (*p* = 0.961). Left entorhinal cortical thickness showed a nominally lower value in AD than in CN participants (*p* = 0.044); however, this finding was not corrected for multiple comparisons and should therefore be interpreted cautiously.

Total hippocampal and amygdala volumes were significantly lower in AD than in CN (6026.8 ± 1049.4 vs. 7699.1 ± 882.8 mm^3^ and 2479.7 ± 549.3 vs. 3301.2 ± 464.3 mm^3^, respectively; both *p* < 0.001). Conversely, total lateral ventricular volume was significantly higher in AD (47,779.6 ± 22,397.1 vs. 32,737.3 ± 16,778.4 mm^3^; *p* < 0.001). These differences persisted after normalization to eTIV.

More specifically, the total hippocampus/eTIV and total amygdala/eTIV ratios were markedly lower in AD than in CN (3.90 ± 0.74 vs. 4.98 ± 0.79 and 1.60 ± 0.39 vs. 2.14 ± 0.39, respectively), whereas the total ventricle/eTIV ratio was higher (30.68 ± 14.03 vs. 21.11 ± 10.88; all *p* < 0.001). Ratios reflecting ventricular enlargement relative to medial temporal structures were also substantially higher in AD, including total ventricle/total hippocampus (8.14 ± 3.95 vs. 4.34 ± 2.38) and total ventricle/total amygdala (20.07 ± 10.16 vs. 10.11 ± 5.37; both *p* < 0.001). Thus, Table 2 demonstrates that the strongest AD–CN differences were observed in medial temporal volumes and ventricular enlargement, whereas most cortical thickness, surface-area, and folding-index measures showed comparatively limited group separation.

**Table 2:** Morphometric comparison between AD and cognitively normal participants.

Parameter	AD (*n* = 153), Mean (SD)	CN (*n* = 365), Mean (SD)	*p* Value	Test
Left hippocampus (mm^3^)	2945.8 (553.0)	3800.3 (435.8)	<0.001	*t*
Right hippocampus (mm^3^)	3081.0 (564.4)	3898.9 (470.2)	<0.001	*t*
Left amygdala (mm^3^)	1161.3 (288.2)	1583.8 (236.9)	<0.001	*t*
Right amygdala (mm^3^)	1318.3 (294.7)	1717.4 (244.1)	<0.001	*t*
Left lateral ventricle (mm^3^)	25,271.1 (12,355.1)	17,259.0 (9191.0)	<0.001	M–W
Right lateral ventricle (mm^3^)	22,508.4 (10,640.1)	15,478.4 (7964.9)	<0.001	M–W
eTIV (mm^3^)	1,562,540.8 (182,505.1)	1,565,682.1 (178,241.7)	0.961	M–W
Left entorhinal thickness (mm)	2.900 (0.501)	2.992 (0.457)	0.044	M–W
Right entorhinal thickness (mm)	2.964 (0.419)	2.991 (0.464)	0.256	M–W
Left entorhinal area (mm^2^)	439.5 (90.1)	430.0 (91.3)	0.206	M–W
Right entorhinal area (mm^2^)	412.3 (80.7)	410.7 (80.5)	0.738	M–W
Left precuneus thickness (mm)	2.12 (0.13)	2.11 (0.14)	0.563	*t*
Right precuneus thickness (mm)	2.11 (0.16)	2.14 (0.15)	0.301	M–W
Left posterior cingulate thickness (mm)	2.20 (0.16)	2.19 (0.15)	0.324	M–W
Right posterior cingulate thickness (mm)	2.19 (0.13)	2.19 (0.16)	0.785	*t*
Left precuneus FoldInd	55.8 (8.2)	55.9 (7.6)	0.797	M–W
Right precuneus FoldInd	56.5 (9.2)	56.3 (9.5)	0.517	M–W
Left posterior cingulate FoldInd	21.6 (4.8)	21.1 (4.9)	0.207	M–W
Right posterior cingulate FoldInd	22.6 (4.0)	22.6 (4.1)	0.909	M–W
Total hippocampus (mm^3^)	6026.8 (1049.4)	7699.1 (882.8)	<0.001	*t*
Total amygdala (mm^3^)	2479.7 (549.3)	3301.2 (464.3)	<0.001	*t*
Total lateral ventricle (mm^3^)	47,779.6 (22,397.1)	32,737.3 (16,778.4)	<0.001	M–W
Total hippocampus/eTIV (×1000)	3.90 (0.74)	4.98 (0.79)	<0.001	*t*
Total amygdala/eTIV (×1000)	1.60 (0.39)	2.14 (0.39)	<0.001	*t*
Total ventricle/eTIV (×1000)	30.68 (14.03)	21.11 (10.88)	<0.001	M–W
Total hippocampus/total amygdala	2.49 (0.39)	2.35 (0.23)	<0.001	*t*
Total ventricle/total hippocampus	8.14 (3.95)	4.34 (2.38)	<0.001	M–W
Total ventricle/total amygdala	20.07 (10.16)	10.11 (5.37)	<0.001	M–W
eTIV/total hippocampus	266.54 (53.69)	206.05 (33.87)	<0.001	M–W
eTIV/total amygdala	662.18 (169.05)	483.88 (89.35)	<0.001	M–W

Data are presented as mean (SD). AD, Alzheimer’s disease; CN, cognitively normal; eTIV, estimated total intracranial volume; FoldInd, folding index; *t*, independent-samples *t*-test; M–W, Mann–Whitney U test. eTIV-normalized ratios were multiplied by 1000 for presentation. A two-sided *p* < 0.05 was considered statistically significant.

### 3.3. Comparison Across CN, MCI, and AD Groups

Three-group analysis demonstrated significant differences in total hippocampal volume, total amygdala volume, total lateral ventricular volume, their eTIV-normalized measures, left entorhinal cortical thickness, and MMSE scores (all *p* < 0.05). Total hippocampal and amygdala volumes progressively decreased from CN to MCI to AD, whereas total lateral ventricular volume increased across these groups (Table 3).

Total hippocampal volume decreased from 7699 ± 883 mm^3^ in CN to 6980 ± 1124 mm^3^ in MCI and 6027 ± 1049 mm^3^ in AD, while total amygdala volume decreased from 3301 ± 464 to 2968 ± 592 and 2480 ± 549 mm^3^, respectively (both *p* < 0.001). Conversely, total lateral ventricular volume increased from 32,737 ± 16,778 mm^3^ in CN to 42,857 ± 22,726 mm^3^ in MCI and 47,780 ± 22,397 mm^3^ in AD (*p* < 0.001). The same graded pattern remained after eTIV normalization. MMSE scores similarly decreased across the three groups (29.1 ± 1.1, 27.4 ± 1.9, and 22.8 ± 2.5, respectively; *p* < 0.001). In contrast, cortical differences were much less pronounced: left entorhinal thickness showed only a modest overall group difference (*p* = 0.027), whereas right entorhinal, left precuneus, and left posterior cingulate thickness did not differ significantly. These results emphasize the intermediate position of MCI for the major volumetric measures while also demonstrating that the cortical measures examined did not show the same clear graded separation.

**Table 3:** Selected morphometric measures across diagnostic groups.

Parameter	CN (*n* = 365)	MCI (*n* = 273)	AD (*n* = 153)	*p* Value
Total hippocampus (mm^3^)	7699 (883)	6980 (1124)	6027 (1049)	<0.001
Total amygdala (mm^3^)	3301 (464)	2968 (592)	2480 (549)	<0.001
Total lateral ventricle (mm^3^)	32,737 (16,778)	42,857 (22,726)	47,780 (22,397)	<0.001
Total hippocampus/eTIV (×1000)	4.98 (0.79)	4.55 (0.89)	3.90 (0.74)	<0.001
Total amygdala/eTIV (×1000)	2.14 (0.39)	1.93 (0.43)	1.60 (0.39)	<0.001
Total ventricle/eTIV (×1000)	21.11 (10.88)	27.78 (15.04)	30.68 (14.03)	<0.001
Left entorhinal thickness (mm)	2.992 (0.457)	2.921 (0.463)	2.900 (0.501)	0.027
Right entorhinal thickness (mm)	2.991 (0.464)	3.004 (0.420)	2.964 (0.419)	0.486
Left precuneus thickness (mm)	2.11 (0.14)	2.09 (0.15)	2.12 (0.13)	0.152
Left posterior cingulate thickness (mm)	2.19 (0.15)	2.17 (0.15)	2.20 (0.16)	0.101
MMSE	29.1 (1.1)	27.4 (1.9)	22.8 (2.5)	<0.001

MMSE, Mini-Mental State Examination; eTIV, estimated total intracranial volume.

### 3.4. Age-, Sex-, and Education-Adjusted Morphometric Analyses

After adjustment for age, sex, and education, significant diagnostic-group effects persisted for total hippocampal volume (F = 145.63, *p* < 0.001), total amygdala volume (F = 131.99, *p* < 0.001), and total lateral ventricular volume (F = 21.65, *p* < 0.001). The corresponding eTIV-normalized measures also remained significantly different across groups for hippocampal volume (F = 80.38, *p* < 0.001), amygdala volume (F = 85.22, *p* < 0.001), and lateral ventricular volume (F = 22.31, *p* < 0.001). In contrast, the previously observed marginal difference in left entorhinal cortical thickness was attenuated after adjustment and was no longer statistically significant (F = 1.85, *p* = 0.158). These results are shown in Table 4.

**Table 4:** Age-, sex-, and education-adjusted morphometric comparisons across diagnostic groups.

Morphometric Measure	*n*	Adjusted Group F	Adjusted *p*	AD–CN Adjusted Contrast
Total hippocampus	789	145.63	<0.001	−1522.4 mm^3^
Total amygdala	789	131.99	<0.001	−767.0 mm^3^
Total lateral ventricle	789	21.65	<0.001	+31.0%
Hippocampus/eTIV ×1000	789	80.38	<0.001	−0.971
Amygdala/eTIV ×1000	789	85.22	<0.001	−0.490
Ventricle/eTIV ×1000	789	22.31	<0.001	+31.7%
Left entorhinal thickness	791	1.85	0.158	−0.074 mm; *p* = 0.112
Right entorhinal thickness	791	0.50	0.607	−0.031 mm
Left precuneus thickness	791	2.06	0.128	+0.005 mm
Left posterior cingulate thickness	791	2.20	0.112	+0.015 mm

### 3.5. Correlations Between Morphometric Measures and MMSE

MMSE scores were positively correlated with total hippocampal volume (*r* = 0.495, *p* < 0.001), total amygdala volume (*r* = 0.462, *p* < 0.001), and eTIV-normalized total hippocampal volume (*r* = 0.421, *p* < 0.001). Total lateral ventricular volume was negatively correlated with MMSE (*r* = −0.256, *p* < 0.001). No significant correlation was observed between left entorhinal cortical thickness and MMSE (*r* = 0.054, *p* = 0.131).

### 3.6. Machine-Learning Classification

In the original six-classifier comparison, Logistic Regression with ElasticNet achieved a mean five-fold cross-validation accuracy of 0.707 ± 0.015, balanced accuracy of 0.712 ± 0.020, macro-F1 score of 0.706 ± 0.017, and ROC-AUC of 0.857 ± 0.007. In the subsequent MMSE-ablation analysis, the corresponding combined model including MMSE achieved a held-out accuracy of 0.698, balanced accuracy of 0.700, macro-F1 score of 0.705, and ROC-AUC of 0.857.

For the primary three-class CN/MCI/AD classification using morphometric, demographic, and clinical/genetic features, Logistic Regression with ElasticNet achieved a mean five-fold cross-validation accuracy of 0.707 ± 0.015, balanced accuracy of 0.712 ± 0.020, macro-F1 score of 0.706 ± 0.017, and ROC-AUC of 0.857 ± 0.007. On the held-out test set, the combined model including MMSE achieved an accuracy of 0.698, balanced accuracy of 0.700, macro-F1 score of 0.705, and ROC-AUC of 0.857. In comparison, the morphometry-only model achieved an accuracy of 0.509, balanced accuracy of 0.505, macro-F1 score of 0.492, and ROC-AUC of 0.704 (Table 5).

Performance was higher for the binary CN-versus-AD task than for the three-class classification task. On the held-out test set, the combined-feature model including MMSE achieved an accuracy of 0.933, balanced accuracy of 0.915, macro-F1 score of 0.919, and ROC-AUC of 0.990. The morphometry-only binary model also showed relatively strong performance, with an accuracy of 0.856, balanced accuracy of 0.823, macro-F1 score of 0.826, and ROC-AUC of 0.901.

The MMSE-ablation analysis further clarified the contribution of cognitive information to classification performance. For the three-class CN/MCI/AD task, adding demographic and genetic variables to the morphometric feature set while excluding MMSE increased held-out accuracy from 0.509 to 0.553, balanced accuracy from 0.505 to 0.545, macro-F1 from 0.492 to 0.531, and ROC-AUC from 0.704 to 0.735. Inclusion of MMSE further increased these metrics to 0.698, 0.700, 0.705, and 0.857, respectively.

A similar pattern was observed for binary CN-versus-AD classification. The combined model excluding MMSE achieved an accuracy of 0.865, balanced accuracy of 0.858, macro-F1 score of 0.845, and ROC-AUC of 0.900, whereas inclusion of MMSE increased accuracy to 0.933, balanced accuracy to 0.915, macro-F1 to 0.919, and ROC-AUC to 0.990. Thus, removal of MMSE was associated with a clear reduction in classification performance, particularly for the three-class task.

**Table 5:** Machine-learning performance on the held-out test set.

Task	Feature Set	Evaluation	Accuracy	Balanced Accuracy	Macro-F1	ROC-AUC
CN/MCI/AD	Morphometry only	Held-out test	0.509	0.505	0.492	0.704
CN/MCI/AD	Combined without MMSE	Held-out test	0.553	0.545	0.531	0.735
CN/MCI/AD	Combined with MMSE	Held-out test	0.698	0.700	0.705	0.857
CN vs. AD	Morphometry only	Held-out test	0.856	0.823	0.826	0.901
CN vs. AD	Combined without MMSE	Held-out test	0.865	0.858	0.845	0.900
CN vs. AD	Combined with MMSE	Held-out test	0.933	0.915	0.919	0.990

The participant-level dataset was stratified into a training set (*n* = 632; 80%) and a held-out test set (*n* = 159; 20%) using a random seed of 42. Performance was evaluated using accuracy, balanced accuracy, macro-F1 score, and ROC-AUC. For the three-class CN/MCI/AD task, ROC-AUC was calculated using a one-vs.-rest approach. The held-out test set was not used during model training or hyperparameter optimization.

## 4. Discussion

In this study, FastSurfer-based morphometric analysis revealed distinct structural alterations across the CN, MCI, and AD groups. The principal findings were significantly lower hippocampal and amygdala volumes and greater lateral ventricular volumes in participants with AD compared with CN individuals. These differences remained significant after normalization to eTIV. Participants with MCI generally showed intermediate values for these volumetric measures. Furthermore, medial temporal volumes were positively associated with MMSE scores, whereas lateral ventricular volume was negatively associated with MMSE. Machine-learning analyses also demonstrated better discriminatory performance for binary CN-versus-AD classification than for the more challenging three-class CN/MCI/AD task. Importantly, the principal hippocampal, amygdala, and ventricular differences remained significant after adjustment for age, sex, and education, whereas the previously marginal left entorhinal cortical-thickness difference was attenuated and no longer statistically significant.

The pronounced hippocampal and amygdala volume reductions observed in AD are consistent with the established vulnerability of medial temporal structures to AD-related neurodegeneration. Hippocampal atrophy is a well-recognized structural feature of AD, reflecting involvement of regions essential for episodic memory. Amygdalar involvement may also occur relatively early in the disease course. Poulin et al. demonstrated prominent amygdala atrophy in early AD and reported an association between amygdala volume loss and clinical disease severity [11]. Our findings are consistent with this observation, as bilateral amygdala volumes were substantially lower in AD than in CN participants.

Raji et al. [12] investigated structural brain volumes in individuals with AD in the context of cognitive reserve and demonstrated volumetric differences in several subcortical structures. Although their study addressed a different clinical question, their findings support the broader relevance of quantitative volumetric MRI for characterizing structural alterations in AD.

The neurodegenerative process affecting the hippocampal formation is not anatomically uniform. The entorhinal cortex and interconnected medial temporal structures are particularly vulnerable during AD progression. In the present study, the marked reduction in total hippocampal volume likely reflects the cumulative macroscopic consequence of neurodegenerative changes involving these interconnected regions. Automated volumetric approaches have similarly demonstrated reductions in subcortical and medial temporal structures in patients with AD [13,14].

An additional finding was the marked enlargement of the lateral ventricles in AD. Ventricular enlargement may represent ex-vacuo expansion secondary to progressive parenchymal loss and therefore provides a complementary measure of neurodegenerative burden. Importantly, differences in hippocampal, amygdala, and ventricular volumes persisted after eTIV normalization, suggesting that these group differences were not attributable solely to interindividual variation in head size. Furthermore, the diagnostic-group effects for total hippocampal volume (F = 145.63, *p* < 0.001), total amygdala volume (F = 131.99, *p* < 0.001), and total lateral ventricular volume (F = 21.65, *p* < 0.001) remained significant after adjustment for age, sex, and education. Significant group effects were also retained for the corresponding eTIV-normalized hippocampal, amygdala, and ventricular measures (all *p* < 0.001; Table 4). These findings indicate that the principal volumetric differences cannot be explained solely by the demographic imbalance among the diagnostic groups.

Among the cortical measures examined, left entorhinal cortical thickness was nominally lower in AD than in CN participants in the unadjusted analysis (*p* = 0.044), whereas most precuneus and posterior cingulate thickness and folding-index measures did not differ significantly between groups. However, the left entorhinal finding was attenuated after adjustment for age, sex, and education and was no longer statistically significant (overall group effect: F = 1.85, *p* = 0.158; adjusted AD-versus-CN contrast: *p* = 0.112). Thus, the unadjusted marginal finding should not be interpreted as evidence of an independent entorhinal cortical difference. Because multiple morphometric parameters were evaluated without formal correction for multiple comparisons, the cortical findings should nevertheless be regarded as exploratory. Previous morphometric studies have suggested that cortical thickness and surface-based measures may complement volumetric markers in characterizing early structural alterations associated with AD [15,16]. Several cohort- and methodology-related factors may have contributed to the limited and asymmetric cortical findings observed in the present study. Notably, 63 of 153 participants with AD (41.2%) had MMSE scores of 24–30, indicating relatively mild clinical severity in a substantial proportion of the AD group and potentially reducing the magnitude of group-level cortical differences. In addition, the multicenter ADNI dataset included images acquired on Siemens, Philips, and GE scanners at both 1.5 and 3 T; although standardized imaging protocols and preprocessing reduce variability, scanner platform and field strength may contribute to residual variability in cortical thickness estimates. FastSurfer has demonstrated high reliability and generally good agreement with FreeSurfer [5], but regional cortical estimates from different automated processing pipelines should not be assumed to be numerically interchangeable. Accordingly, the absence of robust bilateral entorhinal, precuneus, and posterior cingulate differences in this cohort should be interpreted as potentially cohort- and methodology-dependent rather than as evidence against the established cortical involvement of these regions in AD. In contrast, hippocampal and amygdala volume loss and lateral ventricular enlargement showed substantially stronger and more consistent group differences.

Taken together, Table 2, Table 3 and Table 4 indicate that medial temporal volume loss and lateral ventricular enlargement were considerably more robust markers of diagnostic group differences than the cortical measures evaluated in this cohort. The persistence of hippocampal, amygdala, and ventricular differences after eTIV normalization and after adjustment for age, sex, and education argues against head-size variation or these demographic factors as the principal explanation for these findings. Moreover, the progressive decrease in hippocampal and amygdala volumes and corresponding increase in ventricular volume from CN through MCI to AD provide a coherent cross-sectional morphometric pattern. The intermediate MCI values are particularly relevant because they illustrate substantial anatomical overlap between diagnostic groups rather than a sharply separated structural phenotype. However, because the study was cross-sectional, this pattern should not be interpreted as demonstrating within-person progression from MCI to AD.

The association between morphometric measures and cognitive performance further supports their clinical relevance. MMSE scores were positively correlated with total hippocampal and amygdala volumes and negatively correlated with total lateral ventricular volume. Previous studies have similarly reported relationships between cognitive performance and medial temporal structural alterations [17,18,19]. These findings suggest that greater preservation of medial temporal structures is associated with better global cognitive performance. Nevertheless, the moderate magnitude of the observed correlations indicates that cognitive impairment cannot be explained by individual morphometric measures alone. This observation further supports the concept that cognitive status reflects the combined contribution of distributed neurodegenerative changes rather than alteration of a single anatomical structure.

Automated segmentation is particularly advantageous for large neuroimaging datasets such as ADNI, in which manual segmentation is labor-intensive and susceptible to interobserver variability. FastSurfer provides a deep learning-based approach for rapid cortical and subcortical segmentation while generating morphometric outputs compatible with established FreeSurfer workflows. This enables standardized assessment of multiple anatomically relevant regions within large cohorts. Nevertheless, automated measurements remain dependent on image quality, acquisition characteristics, and segmentation performance and should therefore be interpreted within these methodological constraints.

The machine-learning findings provide an additional perspective on the discriminatory information contained in the selected morphometric measures. In the held-out test set, the three-class CN/MCI/AD model incorporating morphometric, demographic, genetic, and cognitive information achieved an accuracy of 0.698, balanced accuracy of 0.700, macro-F1 score of 0.705, and ROC-AUC of 0.857. Performance was higher for binary CN-versus-AD classification, for which the corresponding combined model achieved an accuracy of 0.933, balanced accuracy of 0.915, macro-F1 score of 0.919, and ROC-AUC of 0.990 (Table 5). This difference is plausible because CN and established AD represent more clearly separated ends of the structural and cognitive spectrum, whereas MCI is a heterogeneous intermediate state with substantial overlap with both groups. The higher performance of the binary task therefore illustrates the greater difficulty of resolving intermediate clinical states rather than simply distinguishing clearly separated diagnostic extremes.

The MMSE ablation analysis provides an important qualification to the interpretation of these machine-learning findings. For three-class classification, morphometry alone yielded a held-out macro-F1 score of 0.492 and ROC-AUC of 0.704. Addition of demographic and genetic variables without MMSE produced only a modest improvement (macro-F1 = 0.531; ROC-AUC = 0.735), whereas inclusion of MMSE increased macro-F1 to 0.705 and ROC-AUC to 0.857. A similar pattern was observed for binary CN-versus-AD classification: macro-F1 increased from 0.826 with morphometry alone to 0.845 in the combined model without MMSE and to 0.919 when MMSE was included. These findings indicate that MMSE provides substantial additional discriminatory information, particularly for the three-class classification task. Because MMSE contributes to the clinical characterization of ADNI diagnostic groups, the performance of the combined model should not be interpreted as representing the diagnostic performance of structural MRI morphometry alone. Rather, it reflects integrated discrimination based on morphometric, demographic, genetic, and cognitive information.

Table 5 therefore demonstrates both the discriminatory information contained in FastSurfer-derived morphometry and the incremental contribution of non-imaging predictors. The persistence of meaningful classification performance in the morphometry-only models indicates that structural measures contain diagnostic information; however, the ablation analysis shows that the comparatively high performance of the complete models is substantially influenced by MMSE. The close agreement between cross-validation and held-out test performance should be interpreted as evidence of internal consistency rather than external validation because both the training and test participants were derived from ADNI. Confirmation in independent cohorts therefore remains necessary before clinical application.

Previous studies have demonstrated the potential value of integrating imaging and clinical information in predictive models of AD. Reas et al. [20], using ADNI data, showed that combining quantitative brain MRI, cognitive measures, age, and genetic information improved prediction of progression from MCI to dementia. Our findings are consistent with this multimodal concept, while the present ablation analysis further demonstrates that the contribution of cognitive information should be explicitly distinguished from that of structural morphometry. The present machine-learning models should therefore be regarded as exploratory rather than clinically validated diagnostic tools. Moreover, although a held-out test set was used, it was derived from the same underlying ADNI cohort and therefore represents internal rather than external validation. Independent external validation and evaluation in more heterogeneous clinical populations are required before clinical implementation.

Interpretability is also an important consideration in artificial intelligence-assisted neuroimaging. Böhle et al. [21] demonstrated that explainability methods applied to MRI-based deep learning classification can identify anatomically meaningful regions contributing to AD classification. Such approaches may improve the biological interpretability of machine-learning models. However, explainability analyses were not included in the present study and should therefore be considered a potential direction for future research rather than a finding of the current analysis.

### 4.1. Strengths and Limitations

This study has several strengths. First, it included a relatively large cohort of 791 participants spanning the CN–MCI–AD spectrum, which enabled the evaluation of morphometric differences across diagnostic groups. However, these findings should be regarded as supportive rather than conclusive, given the cross-sectional design and the need for confirmation in independent and longitudinal cohorts. Second, the use of FastSurfer provided an automated and standardized framework for the quantitative assessment of multiple AD-relevant brain regions, including the hippocampus, amygdala, entorhinal cortex, and lateral ventricles. Third, normalization of volumetric measures to eTIV reduced the potential influence of interindividual differences in head size. In addition, adjustment for age, sex, and education demonstrated that the principal hippocampal, amygdala, and ventricular findings were robust to these demographic differences. The simultaneous evaluation of medial temporal atrophy and ventricular enlargement provided complementary measures of AD-related structural changes. Finally, conventional morphometric analyses were complemented by machine-learning models, including a formal MMSE-ablation analysis that allowed the contribution of cognitive information to classification performance to be evaluated explicitly.

Several limitations should also be acknowledged. First, the cross-sectional design precludes evaluation of individual rates of neurodegeneration and prediction of conversion from MCI to AD. Second, ADNI participants are recruited according to standardized research criteria and may not fully represent patients encountered in routine clinical practice, particularly those with substantial neurological, vascular, or systemic comorbidities. Third, ADNI is a multicenter dataset acquired using different scanners, field strengths, and acquisition settings. Although standardized ADNI acquisition protocols and the preferential use of preprocessed, gradient nonlinearity-corrected images were intended to reduce technical variability, scanner manufacturer and field strength were not explicitly modeled as covariates and no additional harmonization procedure was applied. Therefore, residual acquisition-related effects on regional volumetric and cortical measurements cannot be excluded. Although age, sex, and education were included in the adjusted morphometric analyses, residual confounding from other clinical or acquisition-related factors cannot be excluded. The comparatively mild cognitive profile of a substantial proportion of the AD group may also have attenuated cortical group differences. In addition, no formal correction for multiple comparisons was applied across the exploratory morphometric analyses; however, the initially marginal left entorhinal finding did not remain significant after demographic adjustment, further supporting a cautious interpretation of the cortical results. Finally, although the held-out test set was completely separated from model development, it originated from ADNI and therefore does not constitute independent external validation. Furthermore, the MMSE-ablation analysis demonstrated that cognitive information contributed substantially to classification performance; consequently, the performance of the combined models should not be interpreted as reflecting structural MRI morphometry alone. The generalizability of the classification results to routine clinical populations therefore remains uncertain.

### 4.2. Future Directions

Future studies should evaluate longitudinal changes in hippocampal, amygdala, and ventricular measures to determine whether rates of structural change improve prediction of progression from MCI to AD. External validation in independent and more clinically heterogeneous cohorts is particularly important to assess the generalizability of the classification models. Future analyses should also evaluate scanner harmonization, calibration, and the incremental predictive value of individual clinical and imaging feature domains in independent cohorts. Integrating structural MRI morphometry with complementary biomarkers, including amyloid and tau measures, genetic information, and other imaging modalities, may further improve discrimination at earlier stages of cognitive impairment.

### 4.3. Conclusions

FastSurfer-based morphometry demonstrated a clear pattern of medial temporal volume loss and lateral ventricular enlargement across the CN–MCI–AD spectrum. Compared with CN participants, those with AD had substantially lower total hippocampal (6026.8 ± 1049.4 vs. 7699.1 ± 882.8 mm^3^) and amygdala volumes (2479.7 ± 549.3 vs. 3301.2 ± 464.3 mm^3^), whereas total lateral ventricular volume was higher (47,779.6 ± 22,397.1 vs. 32,737.3 ± 16,778.4 mm^3^; all *p* < 0.001); these differences persisted after eTIV normalization. The principal hippocampal, amygdala, and ventricular group effects also remained significant after adjustment for age, sex, and education, whereas the marginal left entorhinal cortical-thickness difference did not. MCI generally showed intermediate morphometric values between CN and AD. MMSE scores were positively associated with total hippocampal and amygdala volumes and negatively associated with lateral ventricular volume. In the held-out test set, the complete three-class model achieved a macro-F1 score of 0.705 and ROC-AUC of 0.857, whereas the corresponding binary CN-versus-AD model achieved a macro-F1 score of 0.919 and ROC-AUC of 0.990. Importantly, removal of MMSE reduced three-class macro-F1 to 0.531 and ROC-AUC to 0.735, demonstrating that cognitive information contributed substantially to the performance of the integrated model. These findings support the potential value of combining automated structural MRI morphometry with demographic, genetic, and cognitive information for quantitative characterization of AD-related differences, while emphasizing that the performance of the combined classifier should not be interpreted as the diagnostic performance of morphometry alone. Because the study was cross-sectional and the models were evaluated within the ADNI cohort, longitudinal and external validation in independent clinical populations is required before clinical application.

## Data Availability

The data used in the present study were acquired from the Alzheimer’s Disease Neuroimaging Initiative (ADNI) database available at the LONI Image and Data Archive (IDA), https://adni.loni.usc.edu/ (accessed on 1 August 2025; see more details about access to the ADNI data: https://adni.loni.usc.edu/data-samples/adni-data/, accessed on 1 August 2025). Registration, confirmation of affiliation and signing the ADNI Data Use Agreement are required for access, and, therefore, a public DOI or direct download link is not provided by the authors. This data were received from the ADNI sources that are available to qualified researchers at the LONI IDA, https://adni.loni.usc.edu/, accessed on 1 August 2025. The authors did not create their own primary clinical database; the analysis was performed based on the secondary ADNI data (ADNI data access granted on 29 April 2025).
